# Genetic variation in placental insufficiency: What have we learned over time?

**DOI:** 10.3389/fcell.2022.1038358

**Published:** 2022-10-14

**Authors:** Li Qing Wang, Icíar Fernandez-Boyano, Wendy P. Robinson

**Affiliations:** ^1^ BC Children’s Hospital Research Institute, Vancouver, BC, Canada; ^2^ Department of Medical Genetics, University of British Columbia, Vancouver, BC, Canada

**Keywords:** placenta, genetics, placental insufficiency, fetal growth restriction (FGR), preeclampsia, miscarriage, chromosomes

## Abstract

Genetic variation shapes placental development and function, which has long been known to impact fetal growth and pregnancy outcomes such as miscarriage or maternal pre-eclampsia. Early epidemiology studies provided evidence of a strong heritable component to these conditions with both maternal and fetal-placental genetic factors contributing. Subsequently, cytogenetic studies of the placenta and the advent of prenatal diagnosis to detect chromosomal abnormalities provided direct evidence of the importance of spontaneously arising genetic variation in the placenta, such as trisomy and uniparental disomy, drawing inferences that remain relevant to this day. Candidate gene approaches highlighted the role of genetic variation in genes influencing immune interactions at the maternal-fetal interface and angiogenic factors. More recently, the emergence of molecular techniques and in particular high-throughput technologies such as Single-Nucleotide Polymorphism (SNP) arrays, has facilitated the discovery of copy number variation and study of SNP associations with conditions related to placental insufficiency. This review integrates past and more recent knowledge to provide important insights into the role of placental function on fetal and perinatal health, as well as into the mechanisms leading to genetic variation during development.

## 1 Introduction

**FIGURE 1:**
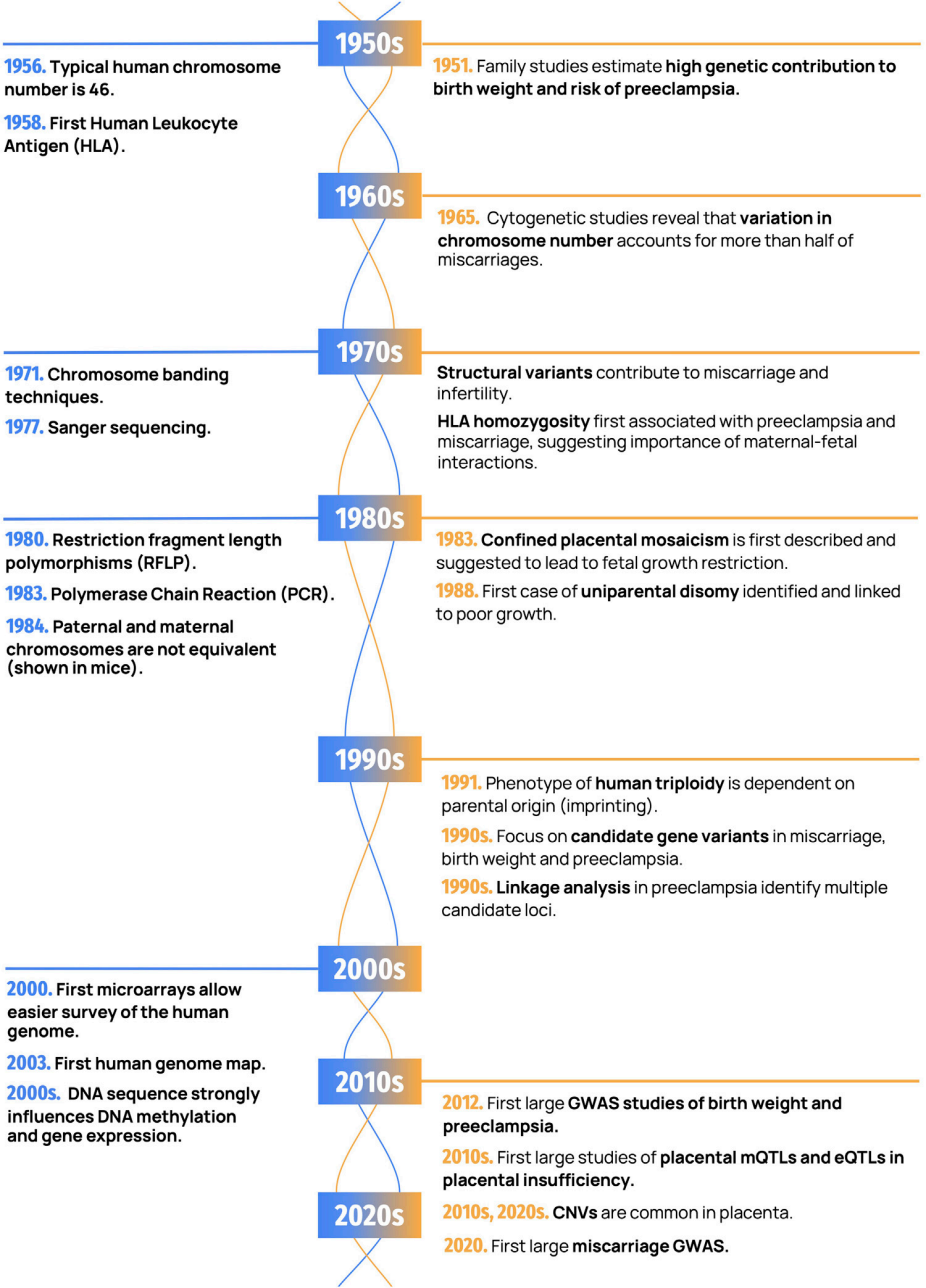
Important discoveries in human genetics and placental genetics. Landmark findings in human genetics are shown in blue (on the left) and placental genetics in orange (on the right).

Human reproduction is a natural process that is fraught with complications. Miscarriage, the spontaneous loss of a pregnancy at <20 weeks, occurs in 15–20% of clinically recognized pregnancies. Preterm birth (PTB), fetal growth restriction (FGR), and preeclampsia (PE) each affect ∼3–10% of viable pregnancies, and these conditions also often co-occur. Many couples that experience adverse pregnancy outcomes seek answers to help them understand what went wrong (Quenby et al., 2021). Given the frequent occurrence of miscarriage and other pregnancy complications, it is not surprising that theories and remedies for promoting fertility and a safe birth have been proposed for millennia (Kuller and Katz, 1994; Mir and Chalak, 2017). The focus has often been placed on women’s behavior as an underlying cause of a failed or complicated pregnancy. However, spontaneous and inherited genetic factors, both maternal, paternal, and their interaction, play a major role in placental health. Our understanding of these genetic underpinnings has increased with advances in genetic technologies over time. While treatments for pregnancy complications are still limited, the diagnosis of genetic causes of placental insufficiency is instrumental in helping families, researchers, and society at large understand how and why pregnancy complications occur. Our goal in this review is to trace the history of advances in genetic techniques and their applications in furthering the understanding of placental insufficiency (Figure 1). We will first outline the epidemiological evidence for the heritability of these conditions, and then discuss the role of chromosome-level changes, followed by a discussion of the role of molecular genetic variation.

## 2 The inception of placental genetics

### 2.1 The nourishing placenta

The placenta has been appreciated as a mediator of fetal nutrition at least as far back as the Hippocrates school in ancient Greece. As early as 340BC, Aristotle aptly described the placental vessels as attaching onto the uterus like the roots of plants, through which the embryo is nourished (Mir and Chalak, 2017). However, the role of the placenta remained surrounded in mystery and myth. It was not until the late 18th and 19th century, that placental structure was described in detail. In 1889, Dutch embryologist A.W.W. Hubrecht was the first to describe “trophoblasts” as cells that form a barrier between the fetus and the mother (Pijnenborg and Vercruysse, 2013). Since then, numerous studies have recognized the role of the placenta in nourishing, as well as protecting the fetus from infection and harmful chemicals through the physical separation of maternal and fetal circulation and molecular processes (Turco and Moffett, 2019).

When the placenta fails to adequately nourish the fetus, pregnancy complications can occur. Placental insufficiency is a general term for the impaired delivery of oxygen and nutrients to the fetus from the placenta (Wardinger and Ambati, 2022). This is commonly attributed to 1) insufficient remodeling of the maternal uterine vessels by invasive trophoblast cells, leading to reduced or intermittent blood flow to the placenta; and/or 2) reduced vascularization (initiation of blood vessels) or angiogenesis (vessel branching) within the placenta (Zygmunt et al., 2003; Burton and Jauniaux, 2004; Alfaidy et al., 2020). Placental malperfusion is also associated with abnormal pathology such as an increase in chorionic villus fibrosis, uteroplacental thrombosis, placental infarcts, and fibrin deposits (Burton and Jauniaux, 2004; Agarwal et al., 2017). Placental insufficiency can lead to miscarriage, preterm labor and birth, FGR, PE, and/or impaired development of fetal organs (Gagnon, 2003; Reus et al., 2013). As low birth weight and PTB are risk factors for many developmental disorders and adverse conditions later in life, placental insufficiency is associated with lifelong health consequences (Gagnon, 2003). These postnatal outcomes may be direct consequences of *in utero* conditions, but they can also in part be explained by the presence of genetic variants that influence placental development.

### 2.2 Establishing a role for inherited factors

The genetic liability of placental insufficiency was first inferred through heritability studies, which evaluate correlation of pathology between related individuals, and segregation analysis, which follows traits within extended pedigrees. The first major study of birth weight heritability was by Karn and Penrose in 1951 based on 1,714 sibships, which estimated that nearly 50% of the variance in birth weight was attributable to shared genetic factors (Karn and Penrose, 1951). A strong genetic influence on birth weight, independent of gestational age, has been confirmed by many subsequent family studies, with the fetoplacental genome contributing most of this effect (Magnus, 1984; Lunde et al., 2007). In an analysis of over 100,000 families from Norway, 31% of the variance in birth weight was estimated to be due to fetal genetic variation (Lunde et al., 2007). Clinical definitions of birth weight include small for gestational age (SGA), defined as birth weight <10^th^ percentile for sex and gestational age, and fetal growth restriction (FGR), previously referred to as intrauterine growth restriction or IUGR, which occurs when low birth weight can be attributed to an underlying pathology.

FGR is commonly found in association with PE, particularly when diagnosed prior to 34 weeks of gestation (Sibai, 2003). Some of the earliest studies suggesting a familial risk for PE came from Humphries and Chelsey *et al.* (O’neal Humphries, 1961; Chesley et al., 1968). In both studies, reviewing hospital records revealed that daughters or granddaughters of women who had experienced PE were at greater risk (two to eight-fold higher) of experiencing PE or eclampsia during their own pregnancies than women without a family history of PE. A genetic contribution to PE has been repeatedly demonstrated, with evidence that both maternal and paternal variants are important (Arngrimsson et al., 1990). Inheritance patterns were initially argued to be consistent with a single gene disorder (Cooper and Liston, 1979; Chesley and Cooper, 1986), but as discussed later in this review, multiple factors including spontaneous chromosome errors in the placenta, genetic mutations, and common variants in both mother and placenta-fetus can all contribute to the susceptibility to PE.

Failure to establish appropriate placental vascularization and maternal spiral artery remodeling early in pregnancy can also underlie spontaneous pregnancy loss. Couples experiencing recurrent pregnancy loss (RPL), defined historically as 3 or more consecutive pregnancy losses, are at greater risk for PTB, FGR and PE in future pregnancies, suggesting at least some overlapping risk factors between RPL and these disorders (Quenby et al., 2021). However, identifying genetic risk factors associated with miscarriage is complicated due to its high incidence, as all women are at risk, and its strong association with maternal age. Miscarriage increases from ∼10% of pregnancies in women in their early 20s to >50% of pregnancies by age 45 (Nybo Andersen et al., 2000; Magnus et al., 2019). Only recently have studies been large enough to demonstrate an increased recurrence risk of miscarriage after a previous miscarriage, though family studies are still largely lacking (Magnus et al., 2019).

In addition to inherited genetic variation, spontaneously arising genetic errors can also contribute to placental insufficiency. These errors may arise during gametogenesis or occur as post-zygotic mutations, the latter of which may play a greater role in the placenta, compared to fetal tissues. However, the relative importance of different genetic factors varies by outcome, as discussed further in this review. Evaluating heritable factors affecting placental insufficiency is also complicated by the heterogeneous nature of the placenta and the influence of environmental exposures such as smoking, alcohol consumption, substance use, and maternal health factors (e.g., obesity, diabetes, and stress) that can influence *in utero* development. Recently, paternal pre-conception obesity has also been implicated in child health outcomes (Campbell and McPherson, 2019), emphasizing that past environmental exposures of both parents is relevant.

## 3 Chromosome-level genetic variation

### 3.1 Counting chromosomes

Following the advent of clinical cytogenetics in the late 1950s, several large studies examined karyotypes from cell cultures of miscarriage specimens, and found that alterations in chromosome number accounted for roughly half of all miscarriages (Carr, 1965; Szulman, 1965). The work of scientists around the world confirmed that a large fraction of pregnancy loss was attributed to spontaneously arising chromosomal alterations especially chromosome trisomy, triploidy, and X monosomy (Carr, 1965; Kajii et al., 1973; Hassold et al., 1978; Warburton et al., 1980). Furthermore, the potential for mosaicism for chromosome abnormalities with a mix of normal and abnormal cells, was also highlighted (Warburton et al., 1978). More recently, studies of leftover embryos from *in-vitro* fertilization (IVF) indicate that at least 50% of human IVF embryos may have chromosomal alterations at the 8-cell stage, indicating that chromosomal variation is remarkably common in human development (van Echten-Arends et al., 2011). Age-related errors in maternal meiosis account for the dramatic increase in the risk of early pregnancy loss with advanced maternal age (Hassold et al., 1980; Hassold and Chiu, 1985). In fact, the calculated rate of chromosomal alterations in miscarriages has increased in recent studies, with estimates as high as 70% (Soler et al., 2017), which may be due in part to the increasing maternal age of pregnant individuals in many populations (Hardy et al., 2016). No environmental factors have been consistently associated with increased risk of pregnancy loss in humans due to chromosomal alterations (Xu et al., 2022).

Molecular techniques have not only improved the detection of smaller chromosomal alterations, but also helped avoid maternal cell contamination, which can plague cell cultures and lead to false 46,XX results (Lathi et al., 2014). Even with advances in technique from conventional karyotyping, trisomy 16, monosomy X, and triploidy remain the most common abnormal karyotypes in recurrent miscarriage specimens (Robinson et al., 2001; Gardner et al., 2011). Trisomy 15, 21, and 22 are also relatively frequent, especially in older women (Robinson et al., 2001). While most complete trisomies are incompatible with life, trisomy confined to the placenta is relatively common and is further discussed in the mosaicism section of this review. Intriguingly, miscarriage also occurs in over 95% of conceptuses with a total absence of one X chromosome (Hook and Warburton, 1983), also known as Turner syndrome, though this condition is generally viable if the fetus survives until birth. This suggests a role for genes that are normally present in two copies in XX and XY individuals, such as those occurring in the pseudoautosomal regions or X-Y homologues.

While trisomy 13, trisomy 18, and trisomy 21 are the only viable non-mosaic autosomal trisomies, embryos with these karyotypes are often lost in the first trimester, possibly due to changes they induce in placental development. For example, studies of cultured cytotrophoblast from trisomy 21 placentae revealed a defect in syncytiotrophoblast formation and a decrease in the synthesis and secretion of syncytiotrophoblast pregnancy-associated hormones such as human chorionic gonadotropin (hCG) (Frendo and Muller, 2001; Pidoux et al., 2007, 2012). These mechanisms could mediate changes in placental function during development. Trisomy 13 has also been associated with occurrence of PE in several studies (Boyd et al., 1987; Dotters-Katz et al., 2017, 2018). Notably, genetic variants near the angiogenic regulator *FLT1*, located on chromosome 13, have been significantly associated with risk of PE (McGinnis et al., 2017).

Given that trisomies of most autosomes have been observed in miscarriages, the overwhelming absence of full trisomies involving chromosomes 1, 11, and 19 reported in the literature is not arbitrary. Chromosome 19 has the highest gene density of all human chromosomes and contains many genes that are crucial for placental function, such as the large placental specific microRNA cluster C19MC (Mong et al., 2020), the Pregnancy Specific Glycoprotein (*PSG*) cluster (Thompson et al., 1990) and the *CGB/LH* gene cluster encoding for chorionic gonadotropin and luteinizing hormone, which may explain the presumed lethality of aneuploidy involving this chromosome. Trisomy 11 may fail implantation due to an imbalance of 11p15.5, which houses a large imprinting region that is important for both placental and fetal development and growth, which is discussed later in this review (Smith et al., 2007). Autosomal monosomies are also largely nonexistent in both liveborns and miscarriages; it is likely that embryos affected by these aneuploidies do not survive long enough on average for pregnancy to be clinically detectable.

Beyond chromosome-level aneuploidies, triploidy is also a commonly observed anomaly in miscarriage karyotypes. In rare cases, triploid conceptuses can survive into the third trimester, though most commonly these pregnancies are spontaneously lost in the first trimester. Studies of triploidy in the human placenta also provided the first evidence for the role of genomic imprinting in the placenta, which is discussed later in this review.

### 3.2 Chromosomes in pieces

Unequivocal identification of individual chromosomes and chromosomal regions became possible easier in the early 1970s with the development of several techniques to stain each chromosome with unique regional banding patterns that could be observed under a microscope. While many structural rearrangements had already been observed with solid staining of chromosomes, techniques such as Giemsa (G-banding) (Barcia, 2007) and quinacrine mustard (Q-banding) (Söhner and Hansson, 2021) allowed for easier interrogation of structural rearrangements, and led to the discovery of more subtle chromosome alterations. This also prompted the establishment of the International System for Human Cytogenetic Nomenclature (ICSN) in 1971, which enabled a standardized description of this chromosomal variation at different levels of resolution (Paris Conference (1971): Standardization in human cytogenetics., 1975). Macroscopic structural variants, typically defined as large genomic alterations involving at least 10Mb, are much less frequent than numerical alterations in spontaneous pregnancy loss. A retrospective analysis of five studies of miscarriages totaling 8,319 cases reported that structural variants were present in 2.9% of first trimester miscarriages (Hardy et al., 2016), which is similar to other studies (Jacobs, 1981; Wu et al., 2021). While rare, certain types of structural variants may deserve particular attention. In 2–4% of couples experiencing RPL, a structural variant is identified in one or both partners (Stephenson, 1996; Maithripala et al., 2018). Carriers of structural variants such as reciprocal or Robertsonian translocations have an increased risk of infertility or pregnancy loss as a consequence of either meiotic arrest (predominantly in male carriers) or an imbalance arising in the conceptus from derivative segregations of these chromosomes (Gardner et al., 2011). However, the severity of phenotypic consequences is dependent on the nature of the rearrangement and the number of chromosomes and breakpoints involved.

### 3.3 Mosaicism in the placenta, more than broken tiles

Until 1983, cytogenetic studies of embryonic and extraembryonic tissue assumed an identical chromosomal complement in both fetus and placenta, given their shared origin in the zygote. That same year, Dagmar Kalousek and Fred Dill were the first to describe the phenomenon of confined placental mosaicism (CPM) (Kalousek and Dill, 1983), the presence of two genetically different cell populations in the placenta, but not the fetus, in a study that sought to determine whether cell lineage contributed to the distribution of chromosome alterations in a human conceptus. They discovered that trisomy was present in the placenta but only diploid cells were found in the fetus in two conceptuses with unexplained fetal growth restriction (FGR), suggesting a possible causal connection (Kalousek and Dill, 1983).

In the same year as Kalousek and Dill identified CPM, *in utero* sampling of placental tissue for prenatal testing, known as chorionic villus sampling (CVS), was first performed by Giuseppe Simoni and Bruno Brambati and used for the diagnosis of trisomy 21 (Brambati and Simoni, 1983). CVS was revolutionary in allowing prenatal testing in the first trimester, since the use of amniocentesis is limited to after 15 weeks of gestation due to safety concerns. However, CVS was subsequently estimated to carry ∼2% risk of miscarriage or other complications (Mujezinovic and Alfirevic, 2007; Tabor et al., 2009). Additionally, in an estimated 1–2% of pregnancies assessed by CVS, aneuploidy was identified that was absent in the cytogenetic analysis of amniotic fluid or cord blood, representing CPM (Ledbetter et al., 1990; Hahnemann and Vejerslev, 1997). More recently, non-invasive prenatal testing (NIPT), which samples cell free DNA in maternal serum, has increasingly been used for prenatal aneuploidy screening. This has brought CPM back into the conversation, as placental trophoblast is the main source of cell-free DNA in maternal plasma for NIPT, and similar to CVS, CPM can lead to discordant NIPT results (Grati et al., 2020; Van Opstal et al., 2020). However, even in the absence of fetal aneuploidy, CPM can be associated with placental insufficiency and poor fetal outcomes (Kalousek et al., 1991; Wilkins-Haug et al., 2006).

The clinical implications of CPM are highly dependent on the degree of mosaicism and the specific abnormality involved, with higher risks to the pregnancy when chromosomes 2, 3, 7, 13, 15, 16, or 22 are involved (Eggermann et al., 2015; Eggenhuizen et al., 2021). In addition, placental mosaicism can be categorized according to the specific placental cell lineage (trophoblast, extraembryonic mesoderm, or both) exhibiting the chromosomal variation, which can be used to infer whether mosaicism arose during mitosis of a diploid conceptus, or during meiosis of a viable aneuploid zygote, with highest risks of adverse outcomes when both lineages are affected (Kalousek and Vekemans, 1996; Benn and Grati, 2021). Overall, pregnancies with higher levels of mosaicism are associated with a higher incidence of PTB, FGR, structural anomalies, and low birth weight (Robinson et al., 1997; Eggenhuizen et al., 2021). Thus, while the placenta is remarkably robust to localized functional impairment caused by non-diploid cell populations - it manages to support fetal growth as long as the level of affected cells is low. Discordant results and a lower predictive performance than for other aneuploidies are also common among NIPT diagnoses of monosomy X. In some cases, this can be explained by monosomy X being confined to the placenta (Serapinas et al., 2016), which can arise in some cases from the rescue of a 45,X conceptus during early development, with somatic nondisjunction leading to a euploid chromosome complement (Rudd et al., 2018).

As previously discussed, trisomy 16 (T16) is one of the most frequent trisomies observed in miscarriage, and it is also arguably the most studied in the context of CPM. For the conceptus to survive, T16 cells must be predominantly confined to the placenta; in some cases, low levels trisomy may also be present in the fetus (Wolstenholme, 1995; Benn, 1998). Even when confined to the placenta, T16 mosaicism (CPM16) is almost always associated with low birth weight for gestational age (Yong et al., 2003; Peng et al., 2021, 16). In addition, PE and fetal cardiac septal defects and hypospadias are significantly more frequent in CPM16 pregnancies than in the general neonatal population (Yong et al., 2003), demonstrating the important role of the placenta in fetal organ development as well as maternal hypertension (Peng et al., 2021).

While outcomes of CPM are well studied from prenatally detected cases, few studies have examined the role of CPM in the general population of SGA babies. In the largest study to date (Del Gobbo et al., 2021), CPM (including multiple cases of CPM16) was found in 12% of SGA placentas in a cohort of 101 SGA pregnancies which included cases with and without associated maternal PE, and 173 controls without CPM. These findings, from a Canadian population, suggest that undetected CPM is the most significant identifiable cause of poor fetal growth, and should particularly be considered in the context of advanced maternal age.

The study of CPM paved the way to a multitude of subsequent findings, which have improved our understanding of the development and function of the placenta. This phenomenon illustrates the crucial role of placental health in fetal growth, which can be affected even if a normal chromosome complement is present in the fetus. In addition, CPM provided evidence that the placenta grows in a clonal fashion, resulting in a patchiness that is important to consider when assessing this organ. Lastly, chromosomal mosaicism is far more common in the placenta than in somatic tissues.

### 3.4 The parent trap: Imprinting and uniparental disomy

In 1984, two independent studies (McGrath and Solter, 1984; Surani et al., 1984) generated mouse embryos with two sets of maternal or paternal chromosomes, respectively. Androgenetic embryos produced only placental tissue whereas gynogenetic embryos produced only embryonic tissue, confirming that the sets of paternally and maternally inherited chromosomes were not functionally equivalent and that both were essential for development. Genomic imprinting is the process underlying this observation, whereby genes are epigenetically modified in the gametes to ensure parent of origin (POE) specific expression after fertilization (Cattanach and Kirk, 1985; Ferguson-Smith et al., 1993). Among several others (Patten et al., 2014), the evolution of genomic imprinting has been linked to the kinship theory, which posits that genes of maternal and paternal origin have conflicting interests (Haig and Westoby, 1989). If imprinting is regulating parental resource allocation, the placenta is the stage where such conflict takes place.

Genomic imprinting in humans is also apparent from studies of triploidy, which were found to differ in phenotype depending on parental origin of the extra set of chromosomes (McFadden and Kalousek, 1991). Digynic triploidy (maternal origin of extra haploid set of chromosomes) is characterized by a small placenta, while diandric triploidy (paternal origin) is associated with a large placenta, often with changes characteristic of a partial hydatidiform mole. As in mice, complete androgenetic conceptuses (hydatidiform moles) in humans result in trophoblast overgrowth and lack embryo formation. Interestingly, mosaicism or chimerism for androgenetic cells confined to the placenta is the main mechanism for placental mesenchymal dysplasia, a rare condition with localized placental findings similar to a partial or complete mole (trophoblastic hyperplasia and edematous villi) and a large placenta (Kaiser-Rogers et al., 2006; Robinson et al., 2007). Placental mesenchymal dysplasia can be associated with a normally developed fetus but FGR is common and affected pregnancies are at high risk for intrauterine fetal death (Pham et al., 2006).

Having a chromosome pair derived from only one parent in a diploid individual is a genetic phenomenon known as uniparental disomy (UPD), which was first systematically studied by Searle and Beechy as well as Cattanach and Kirk in mice (Searle and Beechey, 1978; Cattanach and Kirk, 1985). If imprinted genes are present on the chromosome pair involved in UPD, a distinct phenotypic effect, may result, such as lethality or fetal growth restriction. In the 1980s, Eric Engel hypothesized, based on the high frequency of aneuploidy in humans, that this phenomenon could be a mechanism for human disease through homozygosity for recessive mutations (Engel, 1980). In fact, the first case of UPD identified in humans was in a girl with cystic fibrosis due to homozygosity for a recessive mutation due to maternal UPD7, who was also noted to have short stature (Spence et al., 1988). Shortly after, it was discovered that UPD15 was a mechanism for Prader-Willi syndrome as a consequence of loss of paternal-only expressed genes in 15q11.2 (Nicholls et al., 1989). In the 1990s, an effort was made to identify the phenotypic effects of UPD for each human chromosome (Kotzot and Utermann, 2005). While the effect of UPD is not obvious with certain chromosomes, paternal UPD6 and UPD15 and maternal UPD7, UPD11, UPD15, and UPD20 have been associated with FGR. For instance, maternal UPD7 is a major genetic cause of Silver-Russell syndrome (SRS), seen in ∼5–10% of patients, and is also associated with FGR even in the absence of clinical diagnosis of SRS (Price et al., 1999; Netchine et al., 2007). Chromosome 7 includes the imprinted gene cluster of *PEG1*/*MEST;* this cluster is involved in placental and fetal growth and has recently been shown to be variably imprinted in human preimplantation embryos (Huntriss et al., 2013). However, UPD, including UPD7 (Langlois et al., 1995; Kotzot et al., 2000), is often a consequence of trisomy rescue in CPM, which independently can cause FGR (Robinson et al., 1997). Thus, it can be difficult to separate the effects of trisomy on placental function from the effects of UPD alone.

An imprinting cluster on 11p15.5 is also crucial in the control of fetal growth and has been associated with several pathologies. For instance, loss of DNA methylation at the imprinting control region 1 (ICR1) regulating *IGF2* and *H19* on 11p15.5 is observed in ∼30–60% of patients with SRS, who are commonly born SGA (Gicquel et al., 2005; Netchine et al., 2007; Jacob et al., 2013). Fetal and placental overgrowth can be associated with Beckwith-Wiedemann syndrome (BWS), which is caused by opposite epimutations at ICR1 in 10–20% of cases (Jacob et al., 2013). Interestingly, placental *IGF2* plays an important role in regulating nutrient supply to the fetus in response to fetal demands (Constância et al., 2002; Sandovici et al., 2022).

The discovery that genomic imprinting was typically associated with differential allelic DNA methylation and the subsequent advent of genome-wide DNA methylation arrays facilitated the detection of more imprinted genes. Based on genome-wide methylation profiling it has been observed that many more genes are imprinted in placenta than in somatic tissues (Hanna et al., 2016; Sanchez-Delgado et al., 2016). Interestingly, most placental-specific imprinted regions are maternally imprinted (paternally expressed) as a result of oocyte-derived differential methylation that survives to the blastocyst stage and is maintained in the placenta, and they are often polymorphic (variable between placentas) (Hanna et al., 2016; Sanchez-Delgado et al., 2016). The role of most of these imprinted genes and their impact on placental function is only beginning to be understood (Hanna, 2020).

## 4 Sequence-level variation

### 4.1 HLA and protein polymorphisms as a start to sequence-level variation

Prior to the availability of DNA sequence level polymorphisms, protein variants in blood were identified and used as markers to study human disease and traits. At the time, the most widely studied variants were in the Human Leukocyte Antigen (HLA) genes (Choo, 2007), the first of which was identified by Jean Dausset in 1958 from the detection of alloantibodies in the serum of multiparous women and patients who received transfusions (Carosella, 2009). The *HLA* genes are the most polymorphic system of genes in the human genome, and they are primarily relevant to immune function and transplantation (Bjorkman et al., 1987). Other common protein variants historically used in genetic association studies were the ABO blood groups, G6PD, and HPRT1 (Longo et al., 2002; Li et al., 2021).

The *HLA* region contains over 200 genes, including the highly polymorphic Class I and Class II antigens, which have more than 1,300 variants (Williams, 2001). Early studies used the lymphocytotoxicity technique to assess HLA polymorphisms. Interestingly, it was found that parental *HLA* compatibility was associated with miscarriage (Gerencer et al., 1979) and *HLA* homozygosity with preeclampsia (Redman et al., 1978). These findings were reproduced by multiple other studies (Schacter et al., 1984; Thomas et al., 1985) and were suggested to potentially play a role in maintaining the high polymorphism in this region (Hedrick and Thomson, 1983; Hedrick, 1988). In the 1980s, methods such as restriction fragment length polymorphism (RFLP), polymerase chain reaction (PCR), microsatellite, and automated sequencing opened new avenues for directly determining *HLA* haplotypes. Ober *et al.* in 1998 genotyped 111 Hutterite couples for 14 *HLA* genes, and prospectively followed their pregnancy outcomes. Embryos with paternal and maternal histoincompatibility, i.e., without matching haplotypes, were not rejected by the mother. However, rates of fetal loss were significantly increased when the identity of the paternal and maternal HLA haplotypes matched extensively (Ober et al., 1998). Implantation requires a balance between pro- and anti-inflammatory factors, and a maternal-fetal HLA-discordance may help promote a normal maternal inflammatory response that is conducive to successful implantation (Aggarwal et al., 2019). In the 2000s, *HLA* genotyping was advanced by the use of sequence-specific oligonucleotide probes (SSO) in microarrays (Triche et al., 2014). Genotyping of maternal and fetal *HLA-A, HLA-B, HLA-C, HLA-DRB1,* and *HLA-DQB1* in 258 PE cases and 182 normotensive controls provided further evidence for the association between HLA histoincompatibility and adequate placentation (Triche et al., 2014). It has been postulated that HLA matching between maternal and fetal genotypes influences maternal T cell activity in the placenta, which can be implicated in PE (Darmochwal-Kolarz et al., 2007; van ‘t Hof et al., 2021).

While the classical HLA molecules, HLA-A and HLA-B, are not expressed in placental trophoblast (Moffett and Loke, 2006), HLA-C, -E and -G are expressed by extravillous trophoblasts (EVTs) and have functional roles interacting with other immune factors at the maternal-fetal interface (Kovats et al., 1990; Moffett-King, 2002). HLA-C is most polymorphic of these and directly interacts with maternal uterine Natural Killer (uNK) cells during maternal spiral artery remodeling (Moffett-King, 2002; Vilches and Parham, 2002). There are multiple *KIR* genes that can be classified into the two primary KIR haplotypes A and B, which have inhibitory and activating effects, respectively. The *KIR* genes on these haplotypes differentially interact with two main forms of HLA-C, HLA-C1 and HLA-C2, which are defined by a single polymorphism in the α1 domain of the α helix (Faridi and Agrawal, 2011). Hiby et al. (2004) found that the combination of the “AA” genotype of maternal *KIR* and the “C2” genotype of fetal *HLA-C* was associated with an elevated risk of PE (Hiby et al., 2004). Interestingly, the prevalence of PE was even higher when the placental C2 was paternally inherited. These interactions were also implicated in recurrent miscarriage (Hiby et al., 2010) and the regulation of birth weight (Hiby et al., 2014). It was proposed that increased activity from activating KIRs helps promote EVT simulation and invasion, thus protecting against placental insufficiency (Parham and Guethlein, 2010). Placentation also relies on HLA-G (Gregori et al., 2015), which is thought to help suppress maternal immune response allowing for embryo implantation (Fuzzi et al., 2002). Low maternal levels of soluble HLA-G have been reported in cases of recurrent miscarriage (Zidi et al., 2016) and placental abruption (Steinborn et al., 2003). While there is little polymorphism in the coding region of this gene, variation in the regulatory region that results in reduced expression levels has been observed in PE (Tang et al., 2015).

### 4.2 The candidate gene approach to identifying common gene variants

Candidate gene approaches test for population-level associations with common genetic variants selected based on a postulated biological relevance. Such approaches are most successful in relatively homogenous populations where fewer genetic variants may contribute to a given disorder. Advances in polymorphism detection (RFLPs, PCR-based assays, and eventually, sequencing technologies) enabled scientists to examine candidate genes beyond those in the *HLA* gene family and explore the wider variety of genetic variants that may play a role in placental insufficiency. Some genes examined included those linked to vascular function (e.g., angiotensin), inherited thrombophilias (e.g., factor V), and immune function (e.g., *TLR4*). A comprehensive review of all genes that have been studied with respect to placental insufficiency is beyond the scope of the review, but we provide a few well-studied examples below.

Angiotensin is an important hormone acting as part of the renin-angiotensin system (RAS) to regulate blood pressure. Within the placenta, it has a role in nutrient transport, vascular contractility, and trophoblast invasion (Delforce et al., 2019). In 1993, Ward *et al.* found a PE risk locus at a common polymorphism in the angiotensin (*AGT*) gene promoter (Ward et al., 1993) in a United States population, and validated this finding in a Japanese population. Similar conclusions were reached with a dinucleotide polymorphism in a study involving 22 families from Iceland and Scotland (Soubrier et al., 1993). Notably, an insertion/deletion polymorphism in the angiotensin converting enzyme (*ACE*) gene was also found to be not only associated with PE (Choi et al., 2004), but also strongly associated with idiopathic RPL, as well as PTB and disease severity in preterm infants (Han et al., 2008; Hočevar et al., 2018). The renin-angiotensin system may play a role in placental insufficiency through affecting coagulability, thrombosis, and other pathways.

Pregnancy is a hyper-coagulable state; as such, there is a long history of studying associations with risk factors for thrombophilias in the female partner and adverse pregnancy outcomes including recurrent miscarriage, PE, and FGR. The Leiden mutation in the Factor V gene has been associated with PE, and the mechanism is possibly due to increased thrombosis in the placenta (Dizon-Townson et al., 1996). This mutation has also been implicated in RPL, but the association is only observed in certain populations (Eslami et al., 2020) but not others (Reddy et al., 2019). Overall, thrombophilia may be implicated in pregnancy complications but is unlikely to be their primary cause. Therefore, the screening of such variants in pregnancy is not recommended (Ormesher et al., 2017).

Another gene related to thrombophilia that has been widely investigated is the methylenetetrahydrofolate reductase (*MTHFR*) gene. Variation in the *MTHFR* gene, which codes for a rate-limiting enzyme in the methyl cycle, has been implicated in many health conditions including coronary heart disease and thrombosis. In 2007, Papp *et al.* reported an association between the *MTHFR TT* genotype and both HELLP syndrome and eclampsia, but not PE (Nagy et al., 2007). Similar association studies have also led to conflicting results (Grandone et al., 1997; Sohda et al., 1997; O’Shaughnessy et al., 1999; Laivuori et al., 2000; Kim et al., 2001; Nagy et al., 2007). A meta-analysis of this variant on unexplained RPL confirmed an association in a Chinese population, but not in European populations (Ren and Wang, 2006). False positive associations may be a result of the failure to account for population stratification, which is a common but important limitation in case-control studies. Additionally, population differences in the effect of this variant could be due to other interacting genetic or environmental factors. Currently, the use of *MTHFR* genotyping to evaluate thrombophilia in pregnancy is not recommended by the American Congress of Obstetricians and Gynecologists (Hickey et al., 2013).

In addition to genes involved in vascular function, immune-related genes are also a central focus in placental research due to their key roles in regulating implantation and tolerance at the maternal-fetal interface. Genes involved in innate immunity, such as Toll-like receptor 4 (*TLR4*) (Hirschfeld et al., 2006), oligomerization domain 2 (*NOD2*) (Rijn et al., 2008), and tumor necrosis factor (TNF)-alpha (Molvarec et al., 2008), among others, have been found to be associated with early-onset PE, HELLP syndrome, and severe FGR complicated by PE. However, none of these gene associations has been consistently replicated nor shown to be causal in pregnancy complications.

In summary, many single nucleotide polymorphisms (SNPs) have been reported to be associated with pregnancy outcomes characterized by placental insufficiency. However, many of these associations have not been consistently reproduced (Rull et al., 2012). This may be because of small effect sizes, limited sample sizes, population differences, incomplete information for confounding factors, and disease heterogeneity. In addition, many studies have focused on the maternal genotype without recognizing the potential interaction with fetal genotype during placentation. Nevertheless, these studies and the downstream functional characterization of genes have given insight into some of the basic processes that may underlie placental mediated complications of pregnancy, such as inflammation, vascular processes, and immune responses (Michita et al., 2018).

### 4.3 Identifying rare variants transmitted within individual families: Linkage analysis

As markers became more available throughout the genome, it became possible to scan the genome for predisposing variants without a preconceived hypothesis of biological relevance. There are two main approaches to search for such variants: genome wide-linkage analysis (within family studies), and association studies (population studies). Linkage analysis allows one to identify genes co-segregating with a trait within a family, which relies on the premise that large blocks of the genome are “linked”. This approach is useful when familial patterns of segregation are observed, and the underlying predisposing variants have a high penetrance and low allele frequency in the population. With conditions as common as miscarriage or as heterogeneous as FGR, it can be difficult to identify clear patterns of segregation within a family. However, certain subtypes of PE can in some cases segregate in a familial manner, with many females affected within a single pedigree.

In the early 1990s, the advent of the polymerase chain reaction (PCR) expanded the investigation of genetic variation to microsatellite markers, highly polymorphic tandem repeats (typically 2–6 bp) which could be used to perform genome-wide linkage analyses of diseases with patterns of familial inheritance (Mullis et al., 1986). This approach was used to identify a candidate region for PE on chromosome 4q (Harrison et al., 1997). While highlighting the relevance of linkage analysis for understanding PE, the authors also emphasized the need for replication in an independent cohort. In 1999, screening of 440 microsatellite markers in 124 pedigrees with 343 affected women in Iceland revealed a PE susceptibility locus at 2p13, consistent with the presence of a rare albeit highly penetrant variant responsible for a subset of cases of PE with a very strong familial component (Arngrímsson et al., 1999). This locus was confirmed in a study of 34 families from Australia and New Zealand (Moses et al., 2000). A similar study of 15 families in Finland revealed a susceptibility locus at 2p25 (Laivuori et al., 2003; Majander et al., 2013). In a 2005 linkage analysis of 24 families with at least 2 sisters affected by PE, the 10q22 region was identified. Targeted sequencing in the linked region identified mutations in *STOX1* relevant in the familial inheritance of PE (van Dijk et al., 2005). One risk allele (Y153H) within the *STOX1* gene has been shown to downregulate extravillous trophoblast invasion (van Dijk et al., 2010), and play a functional role in placental vascular remodeling, and may also be associated with PTB and SGA (Dunk et al., 2021). Nonetheless, the role of *STOX1* in PE has not been confirmed in other studies, and may be of minimal impact in the general population (George and Bidwell, 2013).

In summary, pedigree analyses have identified genes linked to the segregation of PE in isolated families, which played an important role in elucidating genes and pathways critical to placental development. Lack of reproducibility is a common issue in linkage studies likely due to limited sample sizes, population differences in the incidence of rare variants, and/or confounding environmental influences. Furthermore, these are generally rare mutations and do not explain PE in the population at large.

### 4.4 Copy number variation in the human placenta: Narrowing down on genes

The development of oligonucleotide array technologies in the early 2000s increased the resolution at which we could study copy number variations (CNVs) in the human genome, and thus identify them as an important contributor to genomic diversity in human populations (Lucito et al., 2003; Sebat et al., 2004). CNVs are defined as DNA fragments of ∼1 kb or larger that are present at a variable copy number relative to the reference genome (Redon et al., 2006). With increased studies and array resolution, it has been estimated that approximately 9.5% of the human genome is comprised of CNVs (Zarrei et al., 2015), making it challenging to distinguish pathogenic from “normal” variation. In the human placental genome, Kasak et al. reported an extensive load of somatic CNVs which were present in higher quantities in normal than complicated pregnancies (Kasak et al., 2015). More recently, Coorens *et al.* used a sequencing-based approach to demonstrate that post-zygotic mutations, including CNVs, are found in the placenta at a higher rate than in any other somatic tissue studied (Coorens et al., 2021). This mutational burden appears to be a part of normal development and may arise by multiple mechanisms unique to the placenta (Robinson and Del Gobbo, 2021). The mutations are often confined to small regions of the placenta due to its clonal pattern of development and are thus unlikely to impact gross fetal/placental development in most cases. Therefore, identifying CNVs with a pathogenic impact in the placenta is particularly challenging.

The role of CNVs in early pregnancy loss and RPL has been studied by several groups. A recent systematic review of these studies identified recurrent submicroscopic deletions at 22q11.21, 2q37.3, and 9p24.3p24.2 associated with spontaneous miscarriage (Wang et al., 2020). These microdeletions have been previously associated with congenital heart disease and neurodevelopmental disorders. Large, likely pathogenic CNVs (>10Mbs) were also identified in 4% of miscarriages. As relevant genes within these CNVs are identified, functional characterization will allow us to better assess the impact of such genes and CNVs in development, and the mechanisms leading to miscarriage (Wen et al., 2015). For example, CNVs affecting *TIMP2,* which is maternally expressed in the placenta, can impact expression and may impair normal development; familial CNVs in this gene have also been associated with RPL (Rajcan-Separovic et al., 2010; Wen et al., 2015).

An increase in CNVs was reported in placentae from pregnancies complicated by PE (N = 10) or FGR (N = 10), compared to gestational-age matched controls (Biron-Shental et al., 2016). In a study of 101 cases of SGA, Del Gobbo *et al.* did not observe a significant difference in total number and cumulative extent of CNVs between placentae associated with SGA *versus* normal growth. However, 3 of 54 (5.7%) euploid SGA placentae contained rare germline CNVs with potential roles in placental function and/or fetal growth that were not present in control placentae. These three CNVs were deemed candidate variants of unknown significance that are potentially pathogenic due to involvement of the *IHNBB*, *HSD11B2, CTCF*, and *CSMD3* genes. A few studies have also reported poor fetal growth in cases where prenatally identified CNVs were confined to the placenta, although counselling in these cases can be difficult (Eckmann-Scholz et al., 2012; Lund et al., 2020, 2021). Together, these findings suggest that the prenatal identification of pathogenic CNVs using NIPT may be clinically relevant and feasible, but a better characterization of placental CNVs first is needed.

### 4.5 Scanning the whole genome: Population association studies

The launch of the Human Genome Project in 1990 was shortly followed by the rapid advancement of sequencing and array technologies during the early 2000s (Durmaz et al., 2015). The development of SNP arrays enabled scientists to interrogate the genome at single-nucleotide resolution for over a million SNPs simultaneously for associations with pregnancy outcomes. These array-based approaches enabled genome-wide association studies (GWASs), which are designed to identify common variants associated with a risk for a given disease or trait (LaFramboise, 2009). Identified variants linked to a risk/trait may be in linkage disequilibrium with causal variants, and the distance between the GWAS variant and the causal variant is usually within 33.5 Kbp (Wu et al., 2017).

While a few GWAS studies have been conducted for PE (Johnson et al., 2012; Gray et al., 2018), the most supported finding is a SNP in *FLT1*, rs4769613, which was identified in a study of 4,380 cases and 310,238 controls (McGinnis et al., 2017). FLT-1 plays a role in angiogenesis in the placenta; elevated levels of FLT-1 may mediate maternal endothelial dysfunction, leading to hypertension (Cerdeira and Karumanchi, 2012). This association was stronger in late-onset than early-onset PE, giving insight into the disease heterogeneity and the importance of subtype stratification.

In 2020, a GWAS for sporadic miscarriage involving 69,054 cases and 359,469 controls revealed a single locus on chromosome 13 (rs146350366) in Europeans for sporadic miscarriage and 3 loci in Europeans for multiple miscarriages (Laisk et al., 2020). The chromosome 13 locus is near *FGF9,* which is implicated in implantation, extravillous trophoblast invasiveness, and trophoblast differentiation, but these findings are yet replicated.

A meta-analysis of GWAS data from 37 studies including 153,781 individuals from various ancestries reported 60 loci associated with birth weight (Horikoshi et al., 2016). These loci were associated with glucose homeostasis and chromatin remodeling. Maternal genetic factors influencing blood pressure have been negatively associated with offspring birth weight (Warrington et al., 2019). However, these alleles only influenced later offspring blood pressure if they were inherited, highlighting the direct effect of fetal genetics. A similar association between maternal genotype and offspring birth weight was observed in Japan; however, this association was mediated by placental weight (Sato et al., 2021). Interestingly, a subset of genetic variants associated with birth weight have also shown parent-of-origin specific effects, in which maternal effects on birth weight were through glucose metabolism and blood pressure regulation (Juliusdottir et al., 2021).

While large GWAS studies have identified new variants that are potentially implicated with placental insufficiency, the genetic factors identified in candidate gene studies with substantial evidence, such as HLA-G and factor V Leiden, have not yet been reproduced. It is possible that GWAS studies have very stringent cut-offs due to multiple testing, such that these associations may not reach the cut-off. Moreover, many GWASs related to placental insufficiency to date only investigate the maternal genotypes, which overlooks the interaction with fetal genotypes that must be considered in placental insufficiency. However, studies involving both genotypes have become available in the recent past, which enabled the distinction between maternal and fetal genetic effects (Warrington et al., 2019). Currently, researchers are studying the combined effect of multiple variants, through an approach known as the polygenic-risk-score (PRS). The placenta has been found to mediate the effect of maternal hypertension genes on offspring birth weight (Sato et al., 2021).

### 4.6 Integrating genomic data with the methylome and transcriptome

While high throughput genotyping techniques led to the identification of several risk loci for placental insufficiency, there was a growing need for methods to prioritize these variants for characterization of functional consequences. A variety of methods have recently emerged, such as the integration of genomic data with the methylomic or transcriptomic data (known as “multi-omics”). In 2006, Bock *et al.* found evidence of an association between DNA sequence and DNA methylation in chromosome 21 (Bock et al., 2006). This formed the early understanding of what was later known as methylation quantitative trait loci (mQTL), SNPs associated with nearby DNA methylation (DNAme). Similarly, SNPs associated with the expression of a nearby gene were termed expression quantitative trait loci (eQTL). In 2017, Peng *et al.* identified abundant eQTLs in the placenta, and suggested their role in mediating the functions of GWAS hits for birth weight, childhood obesity, and childhood BMI (Peng et al., 2017). Subsequent studies reproduced these hits and reported an abundant amount of mQTL in the placenta, the majority of which were related to immune genes, especially *HLA* genes, suggesting the importance of genetic variation in these genes for placental development (Delahaye et al., 2018; Kikas et al., 2019). Only recently, have these mQTL or eQTL been linked to placental insufficiency. For example, Fasil Tekola-Ayele *et al.* identified a list of functional candidate genes for birth weight by integrating genetic, methylation and gene expression data (Tekola-Ayele et al., 2022). The authors suggested that the candidate SNPs may influence birth weight by acting on DNAme and thereby gene expression. The effect sizes of the mQTLs and eQTLs identified were relatively small, and, while multi-omics analyses provide a means to better understand the functional consequences of genetic variants, the challenge of finding strong and reproducible hits remains.

## 5 Challenges of high throughput placental genomics

Recently, high throughput technologies have dominated the field of placental genetics. New variants and pathways related to the various pregnancy complications characterized by placental insufficiency are identified, but the vast majority suffer from poor reproducibility across cohorts. There are some limitations, which must be addressed to facilitate a better understanding of placental insufficiency.

One limitation is sample size and disease heterogeneity. Since the recruitment of pregnant individuals, as well as the collection and processing of placentae require significant resources, studies of the placenta are often limited by a small sample size which can lead false positives due to technical errors, ascertainment biases, or publication biases (*i.e.* results only published when significant). The disadvantage of small sample sizes is compounded by heterogeneity in the trait of interest, such as the presence of evidence for various subtypes of PE and SGA (Leavey et al., 2016; Benton et al., 2018; Roberts et al., 2021). Detailed phenotyping is important for identifying homogeneous study groups. A lack of detailed phenotyping and comprehensive clinical characterization may underpower studies and lead to false negatives.

Additionally, while two genotypes (maternal, and placental-fetal) are involved in studies of the placenta, research has historically focused on the maternal genotype. The contribution of paternal genetics, which can be captured in the fetal genotype, is also important (Galaviz-Hernandez et al., 2018). There is extensive evidence associating the risk of developing PE with paternal family history of PE, limited sperm exposure, and compatibility with maternal genotypes, such as the *HLA* genes (Galaviz-Hernandez et al., 2018). While it can be challenging and costly to obtain both maternal and fetal genotypes, inclusion of both and the study of their interactions may give new insights crucial to understand the mechanisms that play a role in placental insufficiency.

Data sharing is also important as it is resource intensive to collect samples, and this helps to ensure reproducibility and transparency of research (Wilson et al., 2021). Several guidelines have been proposed which include the sharing of complete data and metadata. However, many data repositories lack standardization practices, and a recent meta-analysis found that a significant number of publications still cite a “data available upon request” statement yet fail to respond to such requests consistently. Collective efforts of standardized methods of data collection and sharing may substantially advance future studies of placental insufficiency.

Furthermore, the under-representation of non-white ethnic groups is worth noticing. Prevalence of not only adverse maternal pregnancy outcomes but also long-term consequences of such outcomes are disproportionately elevated in minority groups, such as Black and Indigenous populations (Johnson and Louis, 2022). However, these groups are highly under-represented in genomics research. As genotype frequencies can vary by ancestry, genetic loci that are significant in a population of European ancestry may not apply to other ancestries. In addition to the inclusion of minority groups, the definitions of race, ethnicity and ancestry in the context of genomics research need to be better teased apart.

Lastly, the consideration of environmental factors is relevant because they can interact with genetics. Proper collection of metadata of relevant variables will help better account for confounders in statistical models as well as enable the study of interaction of these factors with genetics.

While it is often costly and time-consuming to address these challenges, the reproducibility crisis suggests that ignoring these issues may cause more harm through the delay in advancing high-quality and inclusive science than can be justified. Collaboration between groups and adherence to recommended guidelines can help overcome these limitations collectively (Wilson et al., 2021).

## 6 Discussion

In summary, the study of placental genetics has evolved considerably over the past century, although the great importance of genetic contributions to placental insufficiency has been consistent. The early appreciation for the role of spontaneously arising chromosomal errors in miscarriage has been expanded to FGR and other pregnancy complications, once it was understood that such abnormalities could be confined to the placenta. Owing to the advances in genomics techniques, scientists studying placental insufficiency have gone from early studies demonstrating a strong inherited predisposition to identifying multiple sequence-level variants that contribute to PE and FGR. While *in utero* exposures such as maternal smoking or diabetes and even paternal preconception exposures also play a role in these conditions, the extensive research demonstrating a role of underlying genetic factors is often overlooked or under-appreciated, and may interact with environmental influences in affection risk.

Furthering the understanding of the genetics of placental insufficiency has implications not only for basic science researchers but also for clinicians. Chromosomal errors such as aneuploidy or structural variation are easy to diagnose, and screening for this in miscarriage samples or placentas associated with FGR can provide more clarity and relieve anxiety for couples. Chromosomal errors are expected to be particularly relevant when the female partner is older, i.e. >37 years of age. Meanwhile, the effects of sequence-level variation are often more subtle and there is a growing challenge to interpret them. Integrative approaches to such data, such as polygenic risk scores, which can predict the probability of disease based on a panel of SNPs, could be used in combination with other clinical markers assessed during pregnancy to improve risk prediction. Aside from the advancement of genotyping technologies, the scientific community has also begun to take notice of the inequities in healthcare, such as racism, which affect pregnancy outcomes and inevitably impact the study of placental genetics. As we move forward in application of genomic tools to the study of placental insufficiency, it is important to include individuals of diverse ancestries and demographic backgrounds to address the inequities in pregnancy health and research, which help us understand the broader picture of the origins of pregnancy complications. This review may help remind us of the history of placental genetics and inspire ideas for future research.
